# *KCNC1* ‐Related Progressive Myoclonus Epilepsy: A Case Report

**DOI:** 10.1002/ccr3.70758

**Published:** 2025-08-04

**Authors:** Mehri Salari, Sara Sadeghzadeh, Masoud Etemadifar

**Affiliations:** ^1^ Shahid Beheshti University of Medical Sciences Tehran Iran; ^2^ Student Research Committee Shahid Beheshti University of Medical Sciences Tehran Iran; ^3^ Faculty of Medicine Isfahan University of Medical Sciences Isfahan Iran

**Keywords:** ataxia, case report, EPM7, *KCNC1*, MEAK, progressive myoclonic epilepsy

## Abstract

*KCNC1*‐related progressive myoclonus epilepsy (EPM7) is a rare disorder causing seizures, myoclonus, and ataxia. The first reported Iranian case highlights the role of genetic testing in diagnosis and potential future treatments, including gene therapy and novel pharmacological approaches.

## 1 Introduction

Progressive myoclonic epilepsy (PME) is a heterogeneous group of epileptic syndromes first introduced in 1903 by Herman Lundborg, a Swedish neurologist. Yet 12 different subtypes are defined for this disease [1]. Progressive myoclonic epilepsy‐7 (EPM7, OMIM #616187), also known as Myoclonus epilepsy and ataxia due to a potassium channel mutation (MEAK), is a recently discovered subtype that is described with rare bilateral (generalized) tonic–clonic seizures, mild cognitive impairment, myoclonia, and ataxia [2]. It is caused by a heterozygous de novo mutation in the *KCNC1* gene on chromosome 11p15.1, leading to the substitution of histidine for arginine [3]. This mutation results in the loss of potassium channel function, subsequently interfering with neurotransmitter release and causing cell death. Fast‐spiking GABAergic interneurons in the neocortex, hippocampus, and Purkinje cells of the cerebellum are affected more than other cells, which is consistent with the symptoms of the disease [4, 5].

Here, to the best of our knowledge, we introduce the first case of EPM7 in Iran, and given the rarity of the disease and the limited data, we provide a summary of the available evidence and possible future treatments in this regard.

## 2 Case History/Examination

**FIGURE 1:**
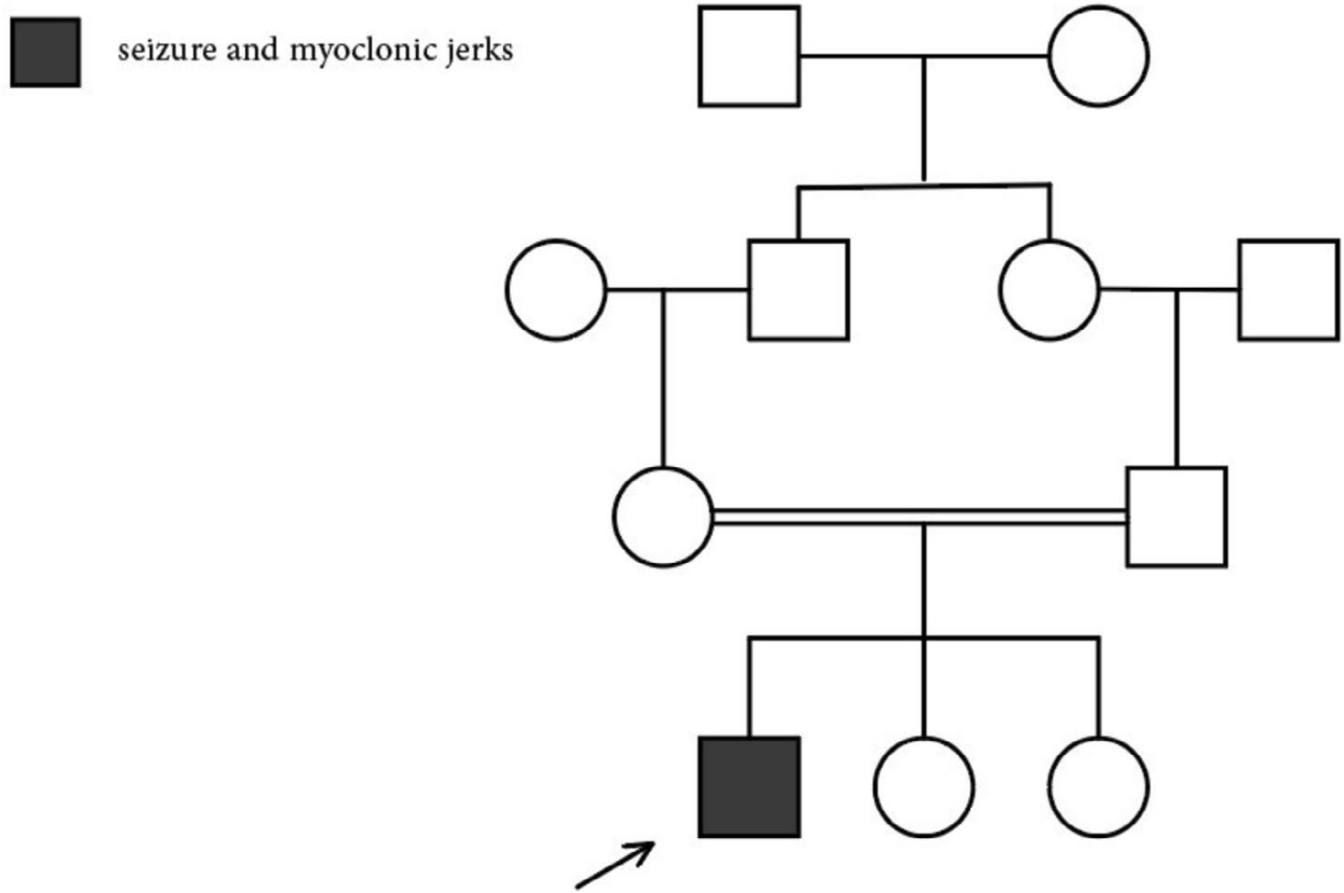
The figure shows the pedigree of the study family. The proband is indicated with an arrow. He has healthy consanguineous parents. His younger siblings did not have any symptoms so far.

This case involves a 27‐year‐old right‐handed Iranian male who presented to our clinic with jerky hand movements and an ataxic gait. He was the first child of healthy consanguineous parents and was born through a normal vaginal delivery without any history of developmental delay (Figure 1). At the age of 15, he had his first episode of generalized tonic–clonic seizures (GTCS). His GTCS were completely controlled with Sodium valproate 500 mg TDS since the first episode. In the past 5 years, he has suffered from progressive imbalance and jerky movements in his hands. He reported no related family history, past drug use, or history of other diseases. His systemic examination was normal. Neurological examination was significant for mild cognitive decline, dysmetria, horizontal gaze‐evoked nystagmus, and truncal ataxia. On limb examination, bilateral myoclonic jerks were seen in the upper limbs. Muscular tone and force were normal. Deep tendon reflexes were normal and plantar reflexes were downward. His gait was ataxic. The patient mentioned neither autonomic nor bladder dysfunction. Other cranial nerves and sensory assessment were also normal (Video 1). Laboratory evaluations such as liver and renal function tests, thyroid function, blood and urine amino acid profiles, and serum vitamin B12 levels were within the normal range. Brain MRI showed mild cerebellar atrophy, and EEG was normal. Whole‐exome sequencing was performed and reported a heterozygous pathogenic variant of the *KCNC1* gene in NM_001112741.2 c.959G>A (p.Arg320His) compatible with EPM7.

**VIDEO 1:** The video shows action‐induced myoclonus in the hands and legs, dysmetria while performing finger to nose task, and clumsiness on the alternative test on the legs. He has a wide‐based gait while walking and action‐induced myoclonus in the axial muscle. Video content can be viewed at https://onlinelibrary.wiley.com/doi/10.1002/ccr3.70758.

## 3 Differential Diagnosis, Investigation, and Treatment

Given the consanguineous parentage and the presenting symptoms, progressive myoclonic epilepsy syndromes (PME) is a primary diagnostic consideration. Based on the patient's history of adolescent‐onset seizures followed by progressive myoclonus and ataxia, EPM7 seems to be the most probable differential diagnosis among other PME subgroups. EPM7 is characterized by action myoclonus, GTCS in adolescence, and cerebellar dysfunction, often with preserved cognition in early stages. The presence of mild cerebellar atrophy on MRI further supports this consideration.

However, other PMEs, including Unverricht‐Lundborg disease (EPM1) and Lafora disease (EPM2), should also be considered [1]. EPM1, resulting from mutations in the *CSTB* gene, typically presents with stimulus‐sensitive myoclonus and ataxia but lacks the early cognitive decline seen in EPM7 and EPM2 [6]. In contrast, Lafora disease, associated with mutations in the *NHLRC1* or *EPM2A* gene, exhibits a more rapid progression and is characterized by dementia, occipital seizures, and the presence of pathognomonic Lafora bodies detectable on skin biopsy [7].

Spinocerebellar ataxias (SCAs) with associated myoclonus or epilepsy, such as SCA19/22 and Dentatorubral‐Pallidoluysian Atrophy (DRPLA), were also evaluated. While the progressive ataxia, myoclonus, and seizures could align with clinical presentations of SCA19/22 [8], the patient's age of onset and lack of a family history of ataxia make this autosomal dominant diagnosis less likely. Additionally, DRPLA can present with ataxia, myoclonus, and seizures, but is typically distinguished by the presence of chorea, dementia, psychiatric manifestations, and a characteristic CAG repeat expansion [9]. The absence of extrapyramidal symptoms, psychiatric symptoms, and a family history of dominant inheritance makes DRPLA an unlikely diagnosis.

Based on normal lab results, genetic tests, and clinical examinations, the patient was diagnosed with EPM7. Sodium valproate 500 mg TDS was continued for GTCS control. To relieve myoclonus, levetiracetam 500 mg TDS and clonazepam 1 mg daily were added. This regimen resulted in a noticeable reduction in myoclonus severity and a relative improvement in the patient's imbalance and jerky movements.

## 4 Discussion

In line with previously reported cases, this case had a normal developmental history [1]. The mean age of the onset of symptoms has been reported to be between 4 and 16 years old, which is compatible with our case [10]. However, myoclonus and jerky movements are mainly known as the first symptoms of the disease [11], similar to the cases reported by Oliver et al. and Barot et al., an episode of generalized tonic–clonic seizure was the first symptom of this case [2, 11]. Thus, the correct diagnosis of this disease could be challenging in the first stages. Seizures are known to be rare and easily controlled, as seen in our patient [1]. Ataxia is a common symptom and is usually an early finding [1, 2]. In this case, the truncal ataxia started after 5 years and had a progressive course. Remarkable improvement of myoclonus and gait with high fever is a common clinical finding in EPM7 cases [1], yet in this case, similar to the case presented by Pedro et al., no notable improvement has been reported [12]. Mild cognitive decline, as in this case, and low range of verbal performance have been reported in most cases, while due to myoclonus, accurate assessment of non‐verbal abilities is not possible (Table 1) [2, 11].

**TABLE 1:** Clinical and para‐clinical findings in previously reported cases.

Study	Gender	Age(year)	First presentation (age)	Seizure types (onset)	Cerebellar signs	Cognitive problem (onset)	EEG finding	MRI finding	Genetic findings	Treatment	Outcome
Barot et al. [2]	Male	18	Unprovoked GTCS [11]	Unprovoked GTCS [11]	Ataxic gait, dysmetria, multifocal action myoclonus	Difficulty with memory retention, fine motor skills [12]	Generalized spike and wave discharges	Mildly prominent right retro‐cerebellar cistern	KCNC1 (p.R320H)	Valproate zonisamide lamotrigine	Symptoms significantly improved with treatment
Kim et al. Case 1 [10]	Male	12	Tremor [9]	Unprovoked GTCS [12]	Dysmetria, myoclonic jerks, ataxic gait	ADHD [7] autistic features	Generalized polyspike and wave complexes	Mild atrophy of the pons and cerebellum	KCNC1 (c.959G>A, p.Arg320His)	Clonazepam, valproic acid	Decrease in frequency and severity of myoclonic jerks, no change in ataxia and tremors, seizures controlled
Kim et al. Case 2 [10]	Male	10	Unprovoked GTCS [9]	Unprovoked GTCS [9]	Dysmetria, myoclonic jerks, ataxic gait	None	Generalized polyspike and focal spikes in the right frontal area	Not performed	KCNC1 (c.959G>A, p.Arg320His)	Valproic acid	Seizures controlled
Mahale et. al [3]	Male	26	GTCS [10]	Febrile seizure (8 months), GTCS [10]	Slurred speech, myoclonic jerks, tremor, ataxic gait, bilateral horizontal gaze evoked jerky nystagmus	Mild deficit in attention, verbal fluency, and memory	Normal awake and sleep records.	Mild cerebellar atrophy	KCNC1 (c.959G>A)	Levetiracetam	Self‐ambulant at 26, myoclonic jerks stopped at 23, seizures controlled
Pedro et al. [12]	Female	29	Jerky movement [10]	Focal epileptic seizures [10] GTCS	Severe generalized action myoclonus, global ataxia, dysarthria	Delay in walking and language	Generalized spike and spike–wave paroxysms	Mild cerebellar atrophy	KCNC1 (c.959G>A)	Valproate	At age 21 was wheelchair‐bound. Seizures were controlled.
Poirier et al. (3 cases) [5]	2 male, 1 female	1–2	None	None	Hypotonia	Speech delay, lability of attention, intellectual disability	Normal	Normal	KCNC1 (c.1015C4T p. (R339))	None	No changes in symptoms
Oliver et al. [11]	10 females 10 males	17–63	Tremor in 14 patients, GTCS in 3 cases, mean onset age was 10	GTCS in 3 cases	Ataxia in 19 cases dysmetria in 12 cases hypotonia in 2 cases	Mild cognitive decline in 11 cases	Generalized polyspike, polyspike‐wave in 11 cases	Global symmetrical cerebellar atrophy in 11 cases, enlarged 4th ventricle, prominent corpus callosum	KCNC1 (c.959G>A; p.R320H)	Valproate zonisamide clonazepam levetiracetam lamotrigine	Valproate was the most effective drug against myoclonus, while Lamotrigine worsened myoclonus
This case	Male	27	GTCS (15)	GTCS (15)	Dysmetria, horizontal gaze‐evoked jerky nystagmus, bilateral myoclonic jerks	Mild cognitive decline	Normal	Mild cerebellar atrophy	KCNC1 (c.959G>A p.Arg320His)	Levetiracetam valproate, clonazepam	Seizures controlled

Abbreviations: ADHD, attention deficit hyperactivity disorder; EEG, electroencephalogram; GTCS, generalized tonic colonic seizure; MRI, magnetic resonance imaging.

Although the disease is inherited in an autosomal dominant pathway, most cases, as this case, are caused by a de novo mutation. Therefore, in clinically suspected cases, genetic testing should be considered despite the absence of a related family history [10, 11]. Interestingly, Kim et al. reported familial cases of EPM7 due to maternal somatic mosaicism with no clinical features [10].

In the case analysis performed by Oliver et al., about half of the patients were wheelchair‐bound in their late teenage mainly due to severe myoclonus. Also, the disease had a nearly stable course in adulthood, and early death has not been reported [11]. In this case, severe truncal ataxia made him wheelchair‐bound in his mid‐twenties.

The most common finding in the EEG reported in previous cases was the preservation of background rhythms along with generalized polyspikes and polyspike waves. Focal occipital epileptiform discharges and spike–wave discharges were also reported in some cases [1, 2, 10, 11]. However, several previous cases similar to this patient have also presented with normal EEGs [3, 5]. This was consistent with our patient's EEG, which showed normal background activity and no photoparoxysmal response.

Regarding treatment, antiseizure medications were the most commonly used option for seizure control in PME cases [13], and valproate had a notable effect in controlling myoclonus in most cases [11]. Deep brain stimulation (DBS) has also been successfully performed in patients with severe disabling myoclonic jerks and tremors [13]. Although currently, the only possible treatment is palliative care, emerging gene therapy and enzyme replacement therapy have opened new horizons for EPM7 patients [12, 13]. The antisense oligonucleotides (ASO) are a new approach targeting encoding mRNAs and have been successfully used in other neurodegenerative diseases such as spinal muscular atrophy [12]. Interestingly, a new small molecule activating compound called RE01 was examined as a direct therapeutic agent in vitro, suggesting that it could be a potential future treatment for the disease [14].

Considering the rarity of the disease and the lack of comprehensive information, we described the first EPM7 case in Iran and reviewed the latest data and possible future treatment methods.

## Author Contributions

**Mehri Salari:** conceptualization, methodology, supervision, writing – review and editing. **Sara Sadeghzadeh:** investigation, methodology, visualization, writing – original draft. **Masoud Etemadifar:** supervision, writing – review and editing.

## Ethics Statement

All authors have read the Journal's position on issues involved in ethical publication and confirm that this article is consistent with those guidelines. This paper is also approved by the ethics committee of Shahid Beheshti University of Medical Sciences. Also, written and informed consent for the online publication of the videos was obtained from the patient.

## Consent

Written consent was obtained from the patient's legal guardians for publication of the case details and accompanying images.

## Conflicts of Interest

The authors declare no conflicts of interest.

## Data Availability

The authors have nothing to report.
